# Ecological succession of the sponge cryptofauna in Hawaiian reefs add new insights to detritus production by pioneering species

**DOI:** 10.1038/s41598-022-18856-8

**Published:** 2022-09-05

**Authors:** Jan Vicente, Molly A. Timmers, Maryann K. Webb, Keisha D. Bahr, Christopher P. Jury, Robert J. Toonen

**Affiliations:** 1 Hawai‘i Institute of Marine Biology, School of Ocean and Earth Science and Technology, University of Hawai‘i at Mānoa, Kāne‘ohe, HI 96744 USA; 2 Pristine Seas, National Geographic Society, Washington, DC 20036 USA; 3 Texas A&M University-Corpus Christi, Corpus Christi, TX 78412 USA

**Keywords:** Biodiversity, Community ecology, Ecosystem ecology, Molecular ecology

## Abstract

Successional theory proposes that fast growing and well dispersed opportunistic species are the first to occupy available space. However, these pioneering species have relatively short life cycles and are eventually outcompeted by species that tend to be longer-lived and have lower dispersal capabilities. Using Autonomous Reef Monitoring Structures (ARMS) as standardized habitats, we examine the assembly and stages of ecological succession among sponge species with distinctive life history traits and physiologies found on cryptic coral reef habitats of Kāneʻohe Bay, Hawaiʻi. Sponge recruitment was monitored bimonthly over 2 years on ARMS deployed within a natural coral reef habitat resembling the surrounding climax community and on ARMS placed in unestablished mesocosms receiving unfiltered seawater directly from the natural reef deployment site. Fast growing haplosclerid and calcareous sponges initially recruited to and dominated the mesocosm ARMS. In contrast, only slow growing long-lived species initially recruited to the reef ARMS, suggesting that despite available space, the stage of ecological succession in the surrounding habitat influences sponge community development in uninhabited space. Sponge composition and diversity between early summer and winter months within mesocosm ARMS shifted significantly as the initially recruited short-lived calcareous and haplosclerid species initially recruit and then died off. The particulate organic carbon contribution of dead sponge tissue from this high degree of competition-free community turnover suggests a possible new component to the sponge loop hypothesis which remains to be tested among these pioneering species. This source of detritus could be significant in early community development of young coastal habitats but less so on established coral reefs where the community is dominated by long-lived colonial sponges.

## Introduction

Understanding species compositional variation across temporal scales is a central goal in community ecology and fundamental for predicting the process of community development under environmental or anthropogenic change^1^. In terrestrial systems, when physical or biological disturbances open space for species to occupy, species composition varies according to the stage of ecological succession within the habitat which is influenced by life history traits among species^2–4^. During early succession, fast growing and well dispersed opportunistic species grow first^5^. However, these species have relatively short life cycles and are eventually displaced by more competitive ones that tend to have lower dispersal capabilities but live longer^6^. Similar patterns of succession are prevalent across temperate and neotropical forests. While the patterns of ecological succession in terrestrial systems have been observed over centuries^2,3,7^ marine systems such as coral reefs have received far less attention^8–10^.

Coral reefs are one of the most biologically rich ecosystems on the planet^11^. The considerable diversity within this ecosystem is primarily attributed to the species occupying the cryptobenthic spaces within the reef framework^12–14^ which are largely overlooked, and can harbor up to 90% of the reef volume and about 50% of reef surface area^15–17^. Sponges are a diverse group of sessile benthic organisms that can comprise up to 73% of the biomass in cryptic habitats^18^ in the Caribbean and 60% of the surface area^19^ within these cryptic habitats in the Red Sea. Sponges are pivotal in sustaining the diversity in these ecosystems through their essential role in nutrient uptake and release^20,21^. Unlike surface-dwelling macrosponges that assimilate dissolved organic matter (DOM) primarily for somatic growth^22^, encrusting and predominantly cryptic sponges use a large portion of their DOM uptake (up to 58%) to fuel rapid cell turnover^23^. Thus, somatic growth for cryptic sponges is assumed to be close to zero since newly formed cells largely replace old cells which are released as detritus^23,24^. This released detritus feeds detritivorous invertebrates thereby fueling the coral reef food web^25^ and making cryptic sponges functionally critical to coral reef ecosystems^26^. Yet despite their functional importance, we know very little about the ecological succession and temporal variability of this important and understudied group of organisms.

Previous work on the succession of Caribbean cryptic reef fauna under ledges and within caves showed that sponges with colonial behavior dominate after pioneering stages^27^. By increasing their persistence over time through lateral expansion, colonial organisms have an advantage over solitary organisms by completely overgrowing space-limited hard substrata^28,29^. While previous studies of cryptobenthic succession provided invaluable insight on colonization and competition of forms, they were limited by taxonomic resolution of Porifera as a phylum. Porifera are notoriously difficult to identify to species, but such identification is essential because groups have distinctive life history traits and physiologies that could influence the succession of these communities. Orders within the most abundant and specious sponge class (Demospongiae) have a variety of growth morphologies^30^, life history traits^31,32^, reproductive strategies^33^, and chemical defense mechanisms against predators^34^ – the latter being a tradeoff with rapid growth^35^. On the other hand, the Calcarea are a small, inconspicuous sponge class^36^ that tend to be more solitary than colonial and can reproduce throughout the year exhibiting high fecundity but are short lived^37–39^. In addition to the taxonomic challenges with identifying sponges, previous studies on cryptobenthic succession have primarily been limited to caves and ledges because these environments could easily be observed and monitored. However, the vast diversity of sponges are hidden deep within the interstices of the reef framework^19^, rendering observational and sampling difficulties. As a result, few studies have targeted the cryptobenthic succession of these functionally important sponges.

Standardized artificial sampling devices, such as Autonomous Reef Monitoring Structures (ARMS), overcome cryptobenthic sampling constraints by providing suitable habitat for cryptobenthic marine invertebrates to colonize. ARMS were explicitly designed as a tiered structure to mimic the three-dimensional complexity of the reef and provide unobtrusive access to these elusive cryptobiota^40–42^. In a recent study targeting Hawaiian sponge diversity, ~ 100 new records were documented from the interstitial spaces of the ARMS, despite field surveys covering more than two orders of magnitude more area (1750 m^2^ of reef habitat) than the ARMS (15 m^2^)^43,44^. The proven success of ARMS in capturing a rich diversity of cryptic sponges, combined with the ease by which the structure is disassembled and reassembled, provides an unprecedented opportunity to evaluate the stages of ecological succession among cryptobenthic sponges.

Here we examine sponge community development over two years in ARMS deployed on an established coral reef habitat resembling the local climax community and within ARMS placed in mesocosms receiving unfiltered seawater from the same reef. We report temporal trends in sponge community composition and investigate whether adaptive strategies or physiological constraints among Porifera groups influence the order in which they occupy cryptic spaces throughout ecological succession.

## Materials and methods

### Site locations

Modified two-tiered ARMS (Figure S1b in Vicente et al. (2021)) were used to examine the temporal recruitment of cryptobenthic sponges in mesocosms and natural reef environments. Sponges were sampled every other month for two years (July 2016-June 2018) from six replicate ARMS placed in mesocosms receiving unfiltered, flow-through seawater from an intake pipe along the adjacent reef and from six replicates attached to the bottom of the intake pipe and 2 m away from the intake opening at the Hawaiʻi Institute of Marine Biology (HIMB) on Moku o Loʻe (Coconut Island). The intake pipe is suspended 1 m above the reef and faces the reef slope. Herein, ARMS recovered from the mesocosm tanks are referred to as “mesocosm ARMS”, and those retrieved from the intake pipe are labeled as “reef ARMS” (Figure S1c in Vicente et al. (2021)).

### Mesocosm flow-through system

The flow-through system at HIMB is fed by alternating seawater pumps with intake lines ~ 2 m deep on the adjoining reef flat, and the active line is switched and flushed every two months to prevent fouling within the pipes. This creates consistent incoming water flow with a residence time of 57 min and allows for an influx of larvae directly sourced from the coral reef adjacent to the reef ARMS. Unfiltered seawater from this system fed six replicate mesocosm tanks (70 L) that were set up to mimic the Hawaiian reef community. Considering a mesocosm tank volume of 70 L and an inflow rate of ~ 1.2 L min^−1^ mean residence time was estimated to be roughly 1 h in all mesocosm tank replicates, well in excess of minimum recommendations^45^. Each mesocosm contained the eight most common Hawaiian reef-building coral species (*Porites compressa*, *Porites lobata*, *Porites evermanni*, *Pocillopora acuta, Pocillopora meandrina*, *Montipora capitata*, *Montipora flabellata* and *Montipora patula*) that collectively account for greater than 95% of coral cover in the state of Hawai‘i^46,47^. These corals were placed on a plastic platform above 2 cm of carbonate sand and gravel, and a collection of 3 pieces of reef rubble (roughly 10–20 cm) were collected from the adjacent reef habitat^48^. Each mesocosm tank also contained herbivorous reef snails (*Trochus* spp.) a juvenile Threadfin butterflyfish (*Chaetodon auriga*), a juvenile Convict tang (*Acanthurus triostegus*), and the two-tiered ARMS placed beneath the coral platform to simulate the cryptobenthic habitat of the living reef matrix at biomass values similar to those reported for Hawaiian reef fishes^49^. In addition to ~ 1.2 L min^−1^ of inflowing unfiltered seawater (turnover), water circulation within each mesocosm tank was generated with a Maxi-Jet Pro propeller seawater pump (~ 4900 L hr^−1^ flow) to closely simulate adjacent reef conditions. Chemical and physical parameters of mesocosm ARMS simulating ambient open reef habitats are reported in Timmers et al. (2021) and Jury et al. (2021) as “control”. Temperature data from both mesocosm and reef habitats were monitored throughout (Supplementary Figure S1). Temperature values of mesocosms were meant to simulate mean values for open reef habitats of Kāneʻohe Bay, Hawaiʻi^48,50^ and averaged 0.64 ± 0.06 °C warmer during 18 out of the 23 months of temperature recordings than the adjoining reef habitat at the HIMB.

### Sponge identification

At each sampling period, modified ARMS were disassembled, photographed with a Nikon D7100 and Nikkor 60 mm lens, and were carefully examined for any newly settled sponge recruits. Sponges showing unique morphological features on each plate were individually photographed, subsampled, DNA barcoded, categorized into solitary or colonial, and classified to species or operational taxonomic units (OTUs) following Vicente et al. (2021). Due to the taxonomic challenges associated with this group^51^, and the limited number of species descriptions that currently exists for the majority of these highly understudied cryptic sponge taxa we used individual OTUs as our proxy for species. A total of 314 sponges were collected and vouchered during this survey. All sequences were deposited in GenBank. Accession numbers pertaining to either COI sequences, 28S rRNA sequences or sequences from both loci were provided for 87 vouchers and the remaining 219 vouchers were identified by matching morphological features to the 87 vouchers with DNA accession numbers (Supplementary Table S1) or the 616 voucher specimens with accession numbers reported in Vicente et al. (2021). A classification table was generated for each OTU following Vicente et al. (2021) (Supplementary Table S2). All OTUs were vouchered with the Florida Museum of Natural History at the University of Florida and the HIMB. In situ images of voucher specimens with the FMNH voucher acronym (UF) (Supplementary Table S1) are publicly available at https://www.invertebase.org/portal/checklists/checklist.php?clid=14&pid=where and http://specifyportal.flmnh.ufl.edu/iz/.

### Monitoring temporal sponge diversity and abundance

Vouchered and barcoded sponges were labeled on high-resolution images of individual modified ARMS plates. Metadata for each sponge included: date, ARMS number, plate number (1 through 3), and side of the plate (top or bottom). Each individual sponge was monitored through time using the initial position of recruits as a reference. Alpha diversity estimates were calculated as the total number of OTUs encountered per ARMS at a given time point. For each ARMS, the abundance of each OTU was based on the presence of that OTU on each side of the three plates resulting in values between 0 and 6. When considering the abundance of multiple or all OTUs on an ARMS, the abundance value could range between 1 and 438 for all 73 OTUs ARMS^-1^.

### Data analysis

Data was analyzed and visualized using *R* v.3.6.3^52^. Observed richness, Shannon and Simpsons indices were calculated using the *estimate_richness* function from the ‘vegan v.2.5-6^53^ package (Supplementary Table S3). We used the *specaccum* function to generate OTU richness rarefaction curves for comparison between habitat types. Mean cumulative differences in sponge abundance and observed diversity were analyzed using parametric (*t.test* function) and nonparametric (*wilcox.test* function) paired t-tests. The ‘nlme v.3.1-151′^54^ package with repeated-measures ANOVA and multilevel modeling were used to compare mean differences in species richness and abundance of sponges across time points using the *lme* function. We tested three models (1) a base linear mixed effect model (*lme* function) with ‘time’ and ‘habitat’ as fixed effects and ‘ARMS number’ as a random effect; (2) the base model with compound symmetry; and (3) the base model with autoregressive lag 1 (corAR1) covariance structure. The autoregressive lag 1(corAR1) covariance structure produced the best model based on an ANOVA model comparison. Residuals were normally distributed (QQ-plot linearity between residuals and quantiles) and randomly distributed (residuals vs number of OTUs and abundance of OTUs). The homogeneity of variance (HOV) assumption for abundance was both visually inspected with box plots of residuals vs. fitted values showing similar variation across residuals^55^ and tested through a Levene’s test (*p* = 0.303). HOV for richness was confirmed visually with boxplots showing similar variation across residuals. Significant Tukey’s post hoc pairwise comparisons using the R package ‘lsmeans’ v.2.30^56^ was used to measure significant differences (*p* < 0.05) in sponge diversity and abundance between habitats at different time points.

Nonmetric multidimensional scaling (NMDS) (*metaMDS* function) was used to plot sponge community composition between time points showing peak (June 2017) and minimum (November 2017) recruitment periods. Bray–Curtis dissimilarity indices were computed (*vegdist* function) on square-root transformed sponge OTU abundance. Permutational analysis of multivariate dispersion (PERMDISP – *betadisper* function) was performed to examine community dispersion of OTUs between habitat types and at time points showing peak and minimum recruitment throughout the two years. Permutational analysis of variance (PERMANOVA – *adonis* function) was used to analyze sponge community composition among habitats and time points based on 1000 permutations. OTUs responsible for significant temporal shifts (*p* = 0.01) within the sponge community composition were identified as vectors and plotted within NMDS plots using the *envfit* function. Nonparametric Kruskal–Wallis tests identified sponge classes or orders responsible for significant shifts (*p* < 0.05) in community composition between the two time points. All figures were generated using the R package ‘ggplot 2’ v3.3.3^57^.

## Results

### Cumulative sponge diversity and abundance in mesocosms and reef habitats

**Figure 1:**
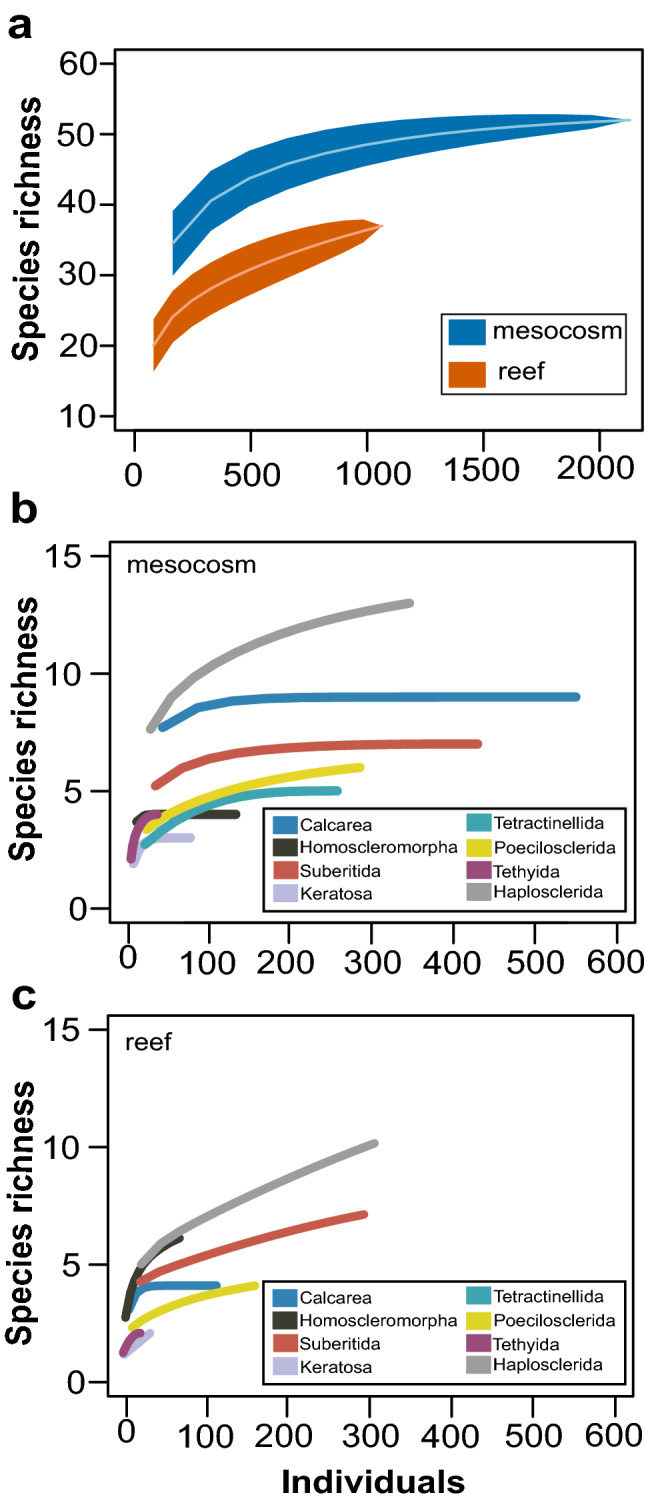
Species rarefraction curves of (**a**) all sponges in mesocosm and reef ARMS, and sponges separated by most abundant groups found in (**b**) mesocosm and (**c**) reef ARMS. Number of individuals refers to sponge recruits encountered at different time points based on presence/absence of sponge OTU within each side of plate from an ARMS. 95% Confidence intervals are included for rarefraction curve in (**a**).

A total of 73 sponge OTUs recruited onto ARMS throughout the 2-year monitoring period (Supplementary Figure S2). Sponge diversity was greatest in class Demospongiae (52 OTUs), followed by Calcarea (13 OTUs) and Homoscleromorpha (8 OTUs). Orders Haplosclerida (18 OTUs), Suberitida (12 OTUs), Poecilosclerida (7), Tethyida (4 OTUs), Tetractinellida (5 OTUs), Dictyoceratida (2 OTUs) and Dendroceratida (2 OTUs) contributed to the overall diversity within Demospongiae (Supplementary Figures S2 and S3). OTUs that were less abundant and not assigned to specific orders within demosponges included Verongimorpha sp. 1 and Heteroscleromorpha sp. 9 (Supplementary Figure S3). Distinct differences in community richness between mesocosm and reef habitats were observed, where 36 OTUs were confined to mesocosms, 18 OTUs were found only on reefs, and 19 OTUs were shared between habitats (Supplementary Figure S2). The proportion of OTUs exhibiting colonial growth was higher on reef ARMS (73%) than mesocosm ARMS (58%) (Supplementary Figure S4). Total richness and abundance of sponge recruits to mesocosm ARMS (diversity = 52 OTUs, abundance = 2130 individuals) exceeded reef ARMS (diversity = 37 OTUs, abundance = 1065 individuals) (Fig. 1; Supplementary Table S3). Higher total richness and abundance of sponge recruits in mesocosms were mainly attributed to sponges in the class Calcarea (diversity = 9 OTUs, abundance = 551 individuals) and Order Tetractinellida (diversity = 5 OTUs, abundance = 257 individuals) (Supplementary Table S3). Calcareous sponges in mesocosms significantly exceeded those on the reef by a mean difference of 3.12 OTUs ARMS^−1^ (t (7.70) = 5.84, *p*_Welch_ =< 0.0001; *p*_Wilcoxon_ = 0.0042) and a mean abundance of 72.50 individuals ARMS^−1^ (t (5.94) = 8.36, *p*_Welch_ =< 0.0001; *p*_Wilcoxon_ = 0.0040). Tetractinellid sponges in mesocosms exceeded those on the reef by a mean difference of 2.17 OTUs ARMS^−1^ (t(5.00) = 5.40, *p*_Welch_ =< 0.0029; *p*_Wilcoxon_ = 0.0023) and 42.83 individuals ARMS^−1^ t(7.70) = 5.84, *p*_Welch_ = 0.0043; *p*_Wilcoxon_ = 0.0028)) (Supplementary Figure S5; Table 1).

**Table 1:** Parametric (Welch Two Sample t-test) and nonparametric (Wilcoxon Rank Sum Test) paired *t* test results comparing cumulative observed diversity and abundance from different sponge groups from reef and mesocosm ARMS.

Test	Taxa group	Classification	Mean difference	Welch two sample t test					Wilcoxon rank
				CI	CI	t	df	*p* value	Sum test *p* value
Diversity	Phylum	Porifera	3.50	− 1.17	8.17	1.85	5.80	0.1157	0.2442
	Class	Calcarea	3.12	1.91	4.43	5.84	7.70	** < 0.0001**	**0.0042**
		Homoscleromorpha	1.16	− 2.50	0.17	− 2.00	8.36	0.0785	0.1113
	Subclass	Keratosa	0.17	− 0.96	0.62	− 0.54	5.00	0.6109	0.5982
	Order	Suberitida	1.17	− 2.63	3.50	− 1.78	9.80	0.1054	0.1380
		Tetractinellida	2.17	1.13	3.20	5.40	5.00	**0.0029**	**0.0023**
		Poecilosclerida	0.00	− 1.28	1.28	0.00	6.76	1.0000	1.0000
		Tethyida	0.33	− 0.61	1.28	0.79	9.41	0.4487	0.4315
		Haplosclerida	0.67	− 0.51	1.85	1.26	9.75	0.2353	0.3396
Abundance	Phylum	Porifera	177.50	142.68	213.32	11.08	9.75	** < 0.0001**	**0.0022**
	Class	Calcarea	72.50	51.22	93.78	8.36	5.94	** < 0.0001**	**0.0040**
		Homoscleromorpha	10.17	− 8.44	28.88	1.25	8.29	0.2446	0.4848
	Subclass	Keratosa	6.84	− 7.81	21.48	1.12	6.46	0.3017	0.7475
	Order	Suberitida	22.34	− 10.02	54.68	1.58	8.44	0.1513	0.1727
		Tetractinellida	42.83	50.56	65.11	4.94	5.00	**0.0043**	**0.0028**
		Poecilosclerida	20.33	− 10.31	50.97	1.59	6.50	0.1583	0.2403
		Tethyida	2.53	− 4.72	8.72	0.71	6.67	0.5014	0.8092
		Haplosclerida	6.17	− 15.99	28.33	0.63	8.94	0.5444	0.7483

Significant values are in bold.

### Temporal distribution of sponge diversity and species abundance

**Figure 2:**
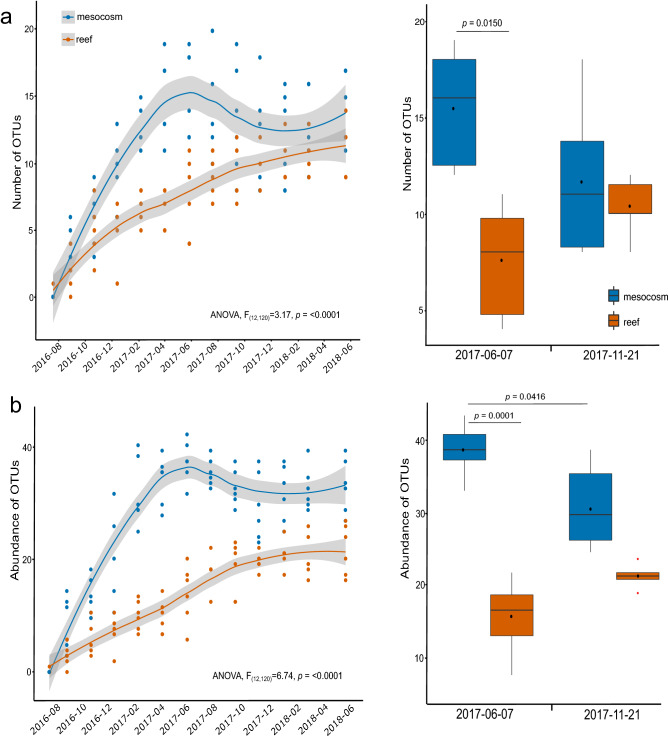
Temporal recruitment of (**a**) sponge OTUs and (**b**) abundance of OTUs settling on mesocosm and reef ARMS in Kāneʽohe Bay. The left panel shows temporal recruitment, monitored bimonthly for approximately 2 years (July 2016 through June 2018). Peak diversity and abundance were observed at 11 months (June 2017) and was lowest at 16 months (November 2017) thereafter. Significant ANOVA results of the interactive effects of time and habitat are reported. Significant differences in diversity/abundance between the two time points (right panel) were observed in mesocosm ARMS as indicated by significant *p* values of Tukey’s post-hoc pairwise comparisons. Significant differences were also observed between mesocosm and reef ARMS. Line graphs are presented with lower and upper pointwise confidence intervals. Boxplots show median values as bars for each habitat; boxes show the upper and lower quartile; whiskers indicate the least and greatest value, excluding outliers. Mean values are shown as black circles and red circles are outliers.

The linear mixed-effects ANOVA showed significant interactive effects of time and habitat on the number of sponge OTUs (*F*_12,120_ = 3.17, *p* =< 0.0001) (Fig. 2a) and abundance of OTUs (*F*_12,120_ = 6.74, *p* =< 0.0001) that recruited onto ARMS (Fig. 2b Table 2). Diversity and abundance of sponges under mesocosm conditions peaked after 11 months during summer (15.5 ± 1.3 (SE) OTUs, 39.0 ± 1.6 OTU abundance) which exceeded recruitment of sponges on the reef by 8 OTUs ARMS^−1^ and exceeded abundance by 24.5 ARMS^−1^ (*p* = 0.0001) (Fig. 2) at the same time point. Following this peak, there was a decline in both diversity and abundance of OTUs in mesocosms, and by month 16, they became much more similar to values observed in the reef ARMS (11.7 ± 1.6 OTUs, 30.7 ± 2.5 OTU abundance). Tukey’s post-hoc pairwise comparisons showed that the decrease in OTU abundance (*p* = 0.0416), but not diversity (*p* > 0.05), of mesocosm ARMS between these two time points was significant (Table 2). In contrast to the mesocosms, diversity and abundance of sponge OTUs that recruited to reef ARMS showed a steady increase with time, reaching a maximum mean diversity similar to that after the mesocosm ARMS declined (11.7 ± 0.9 OTUs) but with statistically insignificant lower mean abundance of 22.7 ± 1.9 throughout the 2 years (Fig. 2).

**Table 2:** Results of the linear mixed effects ANOVA on number of OTUs and abundance by habitat and date.

Linear mixed effects ANOVA	Factor		numDF	denDF	F value	*p* value
Number of OTUs	Habitat		1.00	10	13.76	**0.0040**
	Date		12.00	120	22.62	** < 0.0001**
	Habitat*Date		12.00	120	3.17	** < 0.0001**
Abundance	Habitat		1.00	10	114.04	** < 0.0001**
	Date		12.00	120	36.39	** < 0.0001**
	Habitat*Date		12.00	120	6.74	** < 0.0001**

Post-hoc (Tukey Test)	Pairwise comparison (Habitat * Date)	Estimate	SE	df	t.ratio	*p* value
Number of OTUs	(2017-06-07 mesocosm)—(2017-06-07 reef)	8.00	1.39	10	5.75	**0.0150**
Abundance	(2017–06-07 mesocosm)—(2017-06-07 reef)	24.50	2.40	10	10.21	**0.0001**
	(2017-06-07 mesocosm)—(2017–11-21 mesocosm)	8.33	2.18	120	3.83	**0.0416**

Significant Tukey’s post-hoc pairwise comparisons between habitat and date for peak and minimum recruitment are presented. All pairwise comparisons can be found in Supplementary Table S3 and S4.

Significant values are in bold.

**Figure 3:**
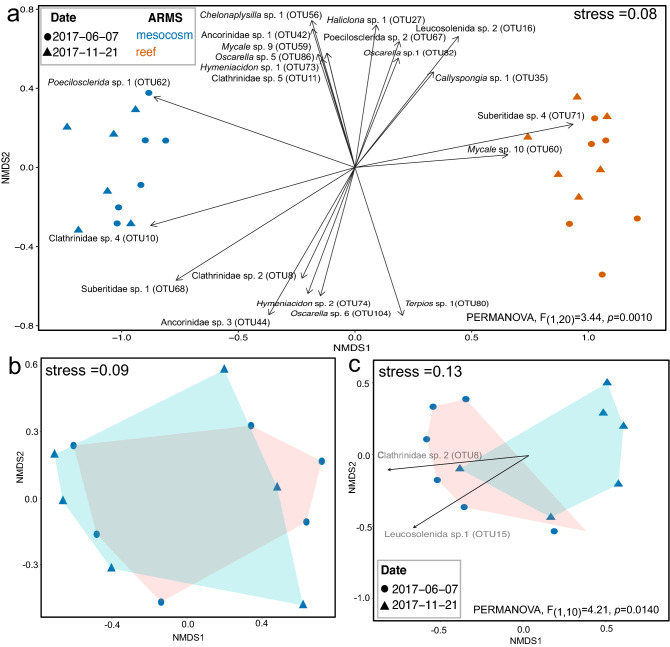
Community composition using multidimensional scaling of Bray–Curtis distances of (**a**) all sponges in mesocosm (in blue) and reef ARMS (in orange) at time points showing peak recruitment at 11 months (June 2017 as triangles) and minimum recruitment at 16 months (November 2017 as circles) thereafter. Community composition of dominant sponge classes (**b**) Demospongiae and (**c**) Calcarea from mesocosm conditions were also analyzed separately. Community composition for the two time points is outlined by blue and pink convex hulls. Significant PERMANOVA results are reported for significant differences in habitat types (**a**) and within the sponge class Calcarea (**c**) in mesocosm ARMS. Vectors in (**a**), and (**b**), show OTUs responsible for significant temporal shifts (*p* = 0.01) within the sponge community composition.

Community composition among Porifera at peak and minimum recruitment differed between reef and mesocosm ARMS (PERMANOVA: *F*_1,20_ = 3.44; R^2^ = 0.49; *p* = 0.0010) (Fig. 3a; Table 3). No significant differences in community composition were observed for the time point main effect or interactive effects between habitat and time point (Fig. 3). Significant differences in community composition between peak and minimum recruitment were driven by Calcareous sponges in mesocosms (PERMANOVA: *F*_1,10_ = 4.21; R^2^ = 0.30; *p* = 0.0160) (Fig. 3b, c; Table 3), and not a result of differences in within-group variability (PERMDISP: *p* > 0.05, Supplementary Table S6).

**Table 3:** Permutational analysis of variance (PERMANOVA) based on Bray–Curtis distances between mesocosm and reef ARMS on time points showing peak and minimum sponge recruitment.

PERMANOVA		Factor	DF	SS	R2	F value	*p* value
Habitat and time points	All sponges	Time point	1	0.16	0.02	1.02	0.3167
		Habitat	1	3.44	0.49	21.87	**0.0010**
		Time point * habitat	1	0.23	0.03	1.46	0.2138
		Residual	20	3.15	0.45	–	–
Within mesocosm	Demospongiae	Time point	1	0.13	0.07	0.75	0.6074
		Residuals	10	1.70	0.93	–	–
	Calcarea	Time point	1	0.44	0.30	4.21	**0.0160**
		Residuals	10	1.04	0.70	–	–

Analysis was also performed on the most dominant sponge classes within mesocosm ARMS. Significance is based on *p* < 0.05.

Significant values are in bold.

**Figure 4:**
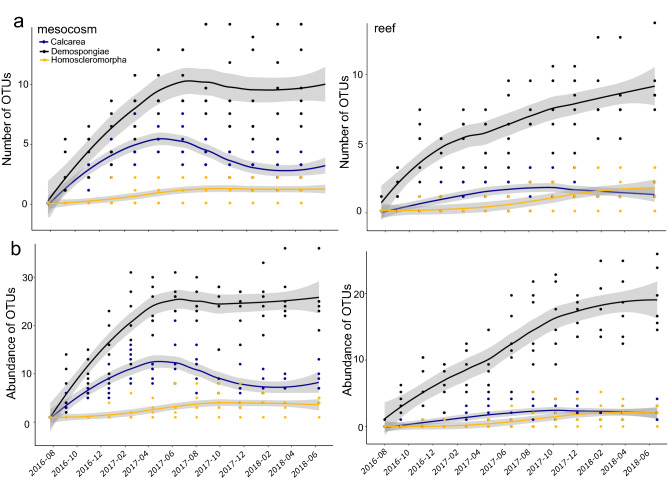
Temporal recruitment of (**a**) sponge OTUs and (**b**) abundance of OTUs settling on mesocosm (left panel) and reef ARMS (right panel). OTUs are presented by sponge classes Calcarea (in purple), Demospongiae (in black) and Homoscleromorpha (in yellow). Line graphs are presented with lower and upper pointwise confidence intervals.

Significant differences through time within the Calcarea were due to a decrease in abundance of Leucosolenida sp. 1 (OTU15), and Clathrinidae sp. 2 (OTU 8) from 11 months during summer to 16 months in early winter (Fig. 3c). The number of Calcareous OTUs and abundance at 11 months (4.8 ± 0.6 (SE) OTUs/11.7 ± 2.1 OTU abundance) significantly exceeded Calcarea at 16 months (2.3 ± 0.5 (SE) OTUs/5.5 ± 1.3 OTU abundance) (chi-square = 5.96, df = 1, *p* = 0.0147 (diversity); chi-square = 5.45, df = 1, *p* = 0.0196 (abundance) Fig. 4; Supplementary Table S7). The Order Haplosclerida also contributed to a decrease in overall sponge diversity from 11 months (3.3 ± 0.3 OTUs/6.8 ± 0.5 OTU abundance) to 16 months (2.0 ± 0.4 OTUs/3.8 ± 1.0 OTU abundance) (chi-square = 4.37, df = 1, *p* = 0.0365 (diversity); chi-square = 5.53, df = 1, *p* = 0.0187 (abundance) Supplementary Figure S6a, Supplementary Table S7). No significant differences between the two time points were observed for other common orders to both habitat types, such as Poecilosclerida (Supplementary Figure S6b), Suberitida (Supplementary Figure S6b) or Tetractinellida (Supplementary Figure S6c; Supplementary Table S7).

## Discussion

The process and stages of ecological succession for the Hawaiian cryptic sponge community differed between ARMS in mesocosms and those on the adjacent reef. Higher recruitment resulted in mesocosm ARMS reaching carrying capacity in one year, at more than double the abundance and richness of sponge OTUs observed on the reef ARMS, which continued to increase in diversity and abundance throughout the two years. Interestingly, the calcareous, haplosclerid and tetractinellid sponges which were responsible for the higher cumulative diversity and abundance observed in mesocosms were rarely observed if not absent on reef ARMS. However, this increased diversity in the mesocosm ARMS was ephemeral; five months after reaching maximum recruitment during the summer, overall richness and abundance in early winter significantly declined due to the reduction in calcareous and haplosclerid sponges. Similar seasonal trends in individual abundance have been reported for the calcareous sponge *Clathrina aurea* in south-eastern Brazil^58^. In contrast, recruitment to the reef ARMS was slower and more consistent, steadily increasing diversity throughout the two-year monitoring period, never reaching an asymptote. Unlike mesocosm ARMS, the reef ARMS were dominated by individuals belonging to the class Homoscleromorpha and orders Poecilosclerida, Tethyida, Keratosa, and Suberitida within the class Demospongiae.

**Figure 5:**
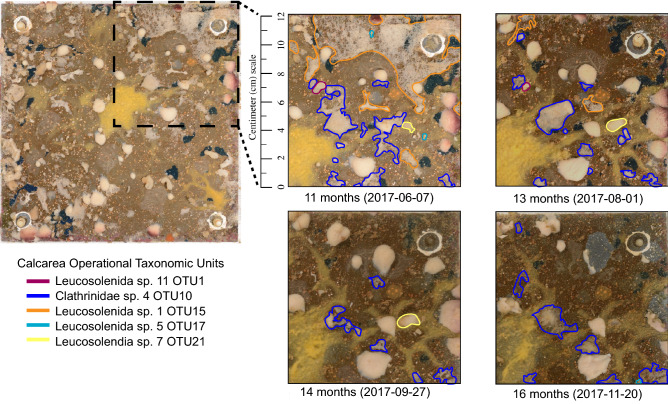
Recruitment of calcareous OTUs from a subsection of a mesocosm ARMS plate through four time points capturing a decrease in diversity and abundance of Calcarea from 2017-06-07 (11 months) through 2017-11-20 (16 months).

Previous studies found that it takes at least three years for cryptic communities in tropical reefs to develop^27^ which might explain why recruitment on reef ARMS did not climax after two years. However, community differences between time points on reef ARMS never transitioned between species as observed in mesocosm ARMS during the same period. Thus, we argue that our inability to observe maximum recruitment after a year on the reef was due to the absence of calcareous and haplosclerid sponges which were only present during pioneering stages of community development. Even though the mesocosm and reef ARMS were ~ 15 m apart, it is possible that the isolation of the mesocosm ARMS from the overall reef could have triggered a true pioneering stage of the cryptobenthic community with the settlement of fast growing but short-lived sponge species. Although mesocosms were established with corals, fish, snails, sand, and rubble to simulate the reef environment^48^, these components do not recreate a fully developed community within the reef matrix. In contrast, reef ARMS were deployed on a mature reef where corals have accounted for 40–60% of benthic cover in the past 20 years^59^. Such long stability in the ecosystems surpasses the time observed for other cryptic communities to reach a climax community. Therefore, we assume that the pioneering stage on this reef has long passed, and a climax cryptobenthic community is already established. Replacement of highly dispersive, solitary and short-lived species by more competitive slower growing and colonial species is characteristic of other systems^5^, including sponges on Caribbean reefs^28^. However, we can discount direct competition as the mechanism by which this replacement occurs because image analyses through time showed that calcareous and haplosclerid sponges were not overgrown or smothered by competitors, but simply disappeared from individual ARMS plates between sampling periods (Fig. 5; Supplementary Figure S7). In addition, the growing behavior of calcareous and haplosclerid OTUs in mesocosm ARMS exhibited solitary form, as opposed to more dominant sponges growing in situ which exhibited colonial behavior and the ability to expand laterally^28^ (Supplementary Figure S8). Thus, we hypothesize that being embedded within a mature reef community allowed recruitment of more competitive, slower growing sponges with colonial growth (Supplementary Figure S4) through lateral expansion to recruit onto reef ARMS.

Without evidence for direct overgrowth of calcareous and haplosclerid sponges, some other differences in biotic or abiotic factors must drive the observed differences in cryptic sponge community succession in this system. Differences in succession between the reef and mesocosm ARMS could be related to a variety of factors that differ between them, such as 1. larval supply, 2. temperature, 3. sedimentation, 4. flow, or 5. nutrition as some of the obvious possibilities. Although our experiment was not set up to test these alternatives, we have some anecdotal evidence to address each. First, it is possible that the composition of larval supply to the mesocosms is different than on the reef, but we used unfiltered seawater pumped directly from the same reef which proved to supply hundreds of species to recruit to the mesocosms over the course of this experiment^50^. Second, as warming has been shown to increase growth and recruitment in some studies^60,61^, the average increase in temperature of 0.64 °C within the mesocosms (Supplementary Figure S1) could have augmented recruitment to the mesocosms. However, Timmers et al. (2021) found that sponge diversity was unaffected by a + 2 °C warming, making it unlikely that warmer temperatures in the mesocosms were responsible for the differences observed here. Third, sedimentation can obstruct recruitment success of sponge larvae^62^ but ARMS from reef and mesocosms had very little sediment accumulation between sampling periods (JV’s personal observations) and any sediment that was present on the ARMS plates in both habitats was cleared during each sampling period. Fourth, water flow has been shown to impact community composition^47^ recruitment^63^, and bleaching susceptibility^45^ of benthic marine organisms. Species richness of benthic invertebrates settling to experimental substrata is expected to increase proportionally to flow rate because of increased delivery of rare species^64^. However, flow rates ensured with water pumps in this study were designed to mimic that of the adjacent reef (~ 10 cm/s), and we see dominance of an entire group of sponges that are not observed on the reef rather than simply adding more rare species as has been observed in previous studies^61,64^. Fifth, the nutritional environment in the mesocosms might differ from that of the reef either because of mass transfer limiting nutrient uptake^65^ or differing particulate or dissolved organic matter (DOM) levels. We supplied unfiltered seawater at a rate sufficient to turn over the entire mesocosm tank volume roughly once per hour in an effort to minimize differences between the reef and mesocosm water chemistry and nutrient content, but we did not measure nutrients. However, periodic filtering of incoming mesocosm water did show that particulates and zooplankton were at the low end of natural reef densities and consistently lower than mean values on the reefs of Kāne‘ohe Bay^48^ suggesting that nutrients may have influenced compositional differences between mesocosm and reef ARMS. Although this list is not exhaustive, none of these factors appear to be obvious drivers of the difference in successional trajectories observed between mesocosms and reef environments.

The debate about whether recruitment limitation, predation or competition is the predominant force in structuring coral reef communities has raged on for decades (e.g.,^66,67^; reviewed by Hixon^68^. Signs of predation within cryptic coral reef communities are rare^28^ as cryptic habitats are known to provide physical protection for palatable sponges from spongivorous fish, cowries, and turtles^69–74^. ARMS provide only 1 cm spacing between plates, similarly, limiting predation from these organisms. Unfortunately, little is known about the species composition or trophic guilds of the cryptic coral reef communities which may harbor a multitude of sponge predators^73–75^. However, some caridean shrimp, polychaetes, and nudibranchs are known to consume sponges but none were observed in our study^76,77^. It is possible that high local densities of invertebrate spongivores on the natural reef consumed the calcareous and haplosclerid sponges, but such predation pressure would need to be extreme to eradicate nearly every individual before they could be sampled from our experimental reef ARMS. Even if these invertebrate predators consume small calcareous or haplosclerid sponges, predation seems an unlikely explanation for the difference between the mesocosm and reef ARMS because we observe a seasonal die-off of these small solitary sponges in the mesocosms which must be free of these predators if that were to explain why we see these sponges in only the mesocosm ARMS. Thus, we see no direct evidence of competition or predation structuring these sponge communities, and do not expect that larval supply differs fundamentally between the mesocosms and the adjacent reef from which our seawater is drawn. This leaves open the important question of which processes drive the difference in primary succession observed for cryptic sponges between reef and mesocosm ARMS as a fertile topic for further research.

Regardless of the factors underlying the observed difference between habitats, our results not only show strong evidence for stages of succession among sponge species with distinctive physiologies colonizing these uninhabited substrates but have important ecological implications for the cryptic community. Short life cycles of calcareous and haplosclerid sponge OTUs during early succession raise the hypothesis that detritus production derived from dead sponge tissue adds an important component to the budget of particulate organic matter (POM) via the sponge-loop. Detritus produced through the sponge-loop was originally reported as a result of rapid turnover of sponge cells^24,78,79^ that feed invertebrate detritivores like brittle stars^25,80^. In light of this discovery, other mechanisms of detritus production from sponges have been hypothesized to contribute to the sponge loop, such as predation of massive sponges which convert DOM into biomass^22,81^. Brandl and colleagues^82^ likewise argue that high productivity and turnover of small cryptobenthic fishes fuel ecosystem function and support the coral reef food web for larger, better known reef fish. Here we propose detritus production in cryptic communities from rapid turnover of these small-bodied solitary sponges could influence nutrient trophodynamics on young, developing Hawaiian coral reef habitats. For example, diversity and abundance between Calcarea and Haplosclerida accounted for 42% of total sponge diversity and abundance under mesocosm conditions before die-off. Death from such a dominant group of sponges in unestablished coral reef communities provide a significant source of available POM for detritovores during early stages of succession. However, POM production from sponge death is less likely to occur in developed coral reef communities where solitary sponges are unable to compete with established colonial species. But to verify this contribution, future studies should evaluate DOM uptake in these pioneering species, while simultaneously calculating their sponge biomass to determine the amount of detritus produced as a result of dead sponge tissue in relation to the entire POM pool of the reef.

Historically, cryptobenthic sponges have been thought of as a competitive phylum that appears only after pioneering stages of ecological succession in coral reef habitats^27,28,83^. However, previous studies have been limited by their inability to capture cryptic diversity deep within the reef matrix, monitoring succession of the reef matrix commuinty in an entirely undeveloped community isolated from coral reef conditions, and by low taxonomic resolution of sponges as a group. Using ARMS in our study and species identification of sponges as a single group subsampled on a temporal scale through barcoding and microscopy overcomes these early limitations. Although it is difficult to estimate complete diversity of sponges in our study considering that cryptic diversity could exist within samples exhibiting the same morphospecies^84^, our data show that the sponge community is diverse, and that this diversity contributes to different stages of ecological succession with some species being early colonists, and others being more competitive throughout community development. ARMS on the reef show trends much more similar to previous work, with some species being early colonists in undeveloped communities, and others being more competitive throughout development in more mature communities; whereas mesocosm ARMS highlight pioneering sponge species (i.e., Calcarea and Haplosclerida) during early stages of succession in undeveloped communities. These early successional sponges might play a previously unappreciated role by contributing to the particulate organic matter budget during the formation of coral reefs. Such processes are critical for maintenance of reef biodiversity throughout early reef development and understanding the contribution of individual species in this rich community is essential to manage and conserve coral reef productivity and biodiversity through space and time.

## Supplementary Information

Supplementary Figures.

Supplementary Tables.

## Data Availability

All samples were vouchered with the Florida Museum of Natural History at the University of Florida and at HIMB. Voucher metadata are publicly available at https://www.invertebase.org/portal/checklists/checklist.php?clid=14&pid=6. Accession numbers pertaining to either COI sequences, 28S rRNA sequences or sequences from both loci were provided for 87 vouchers (Supplementary Table S1). All sequences were deposited in GenBank under accession numbers: MW059040, MT586742, MW059078, MW059081, MW059070, MW143252, MW143254, MW059096, MW059097, MW059108, MW059101, MW059043, MW059071, MW059080, MW059082, MW144988, MW144983, MW059085, MW144984, MW059088, MW059083, MW144975, MW059084, MW059049, MW059063, MW059059, MW059107, MW059094, MW059055, MW059045, MW059053, MW016124, MW016135, MW016094, MW016241, MW016128, MW016216, MW016202, MW016148, MW016118, MW016161, MW016232, MW016229, MW016233, MW016172, MT452531, MW016374, MW016138, MW01630, MW016174, MW016178, MW016205, MW016209, MW016245, MW016123, MW016284, MW016253, MW016210, MW016218, MW016372, MW016129, MW016069, MW016146, MW016304, MW016307, MW016150, MW016211, MW016246, MW016206, MW016061, MW016251, MW016163, MW016090, MW016176, MW016344, MW016060, MW016342, MW016255, MW016331, MW016166, MW016343, MW016275, MW016153, MW016066, MW016184, MW016300, MW016288, MW016347, MW016256, MW016353, MW016257, MW016354, MW016373, MW016260, MW016261, MW016228, MW016259, MW016247, MW016289, MW016248, MW016125, MW016059, MW016324, MW016320, MW016325. Raw data showing distribution of sponge species in mesocosm and reef conditions throughout the 2-year monitoring period are provided in Supplementary Table S8.
