# Supplementary material for: Ecological succession of the sponge cryptofauna in Hawaiian reefs add new insights to detritus production by pioneering species
Source: Sci Rep. 2022 Sep 5;12:15093. doi: 10.1038/s41598-022-18856-8 (PMC9445044; doi:10.1038/s41598-022-18856-8)
Supplement: Supplementary file 1 — Supplementary Figures. [file 41598_2022_18856_MOESM1_ESM.docx]

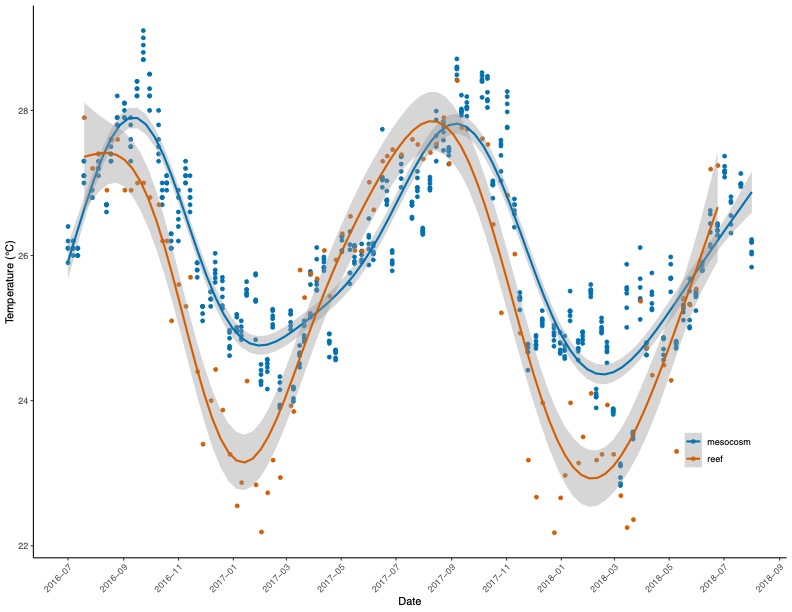


**Supplementary Figure S1**. Temperature values at Moku o Loʽe, Kāneʽohe Bay on reef and mesocosm habitats simulating offshore temperature reef conditions throughout a two-year period. Daily temperature readings on reef conditions were provided by the Pacific Islands Ocean Observing System (PacIOOS) for the Moku o Loʻe weather station which has a temperature probe within 20 m from ARMS in situ. Six replicate readings provided here were measured at 1200 hr from individual mesocosms.


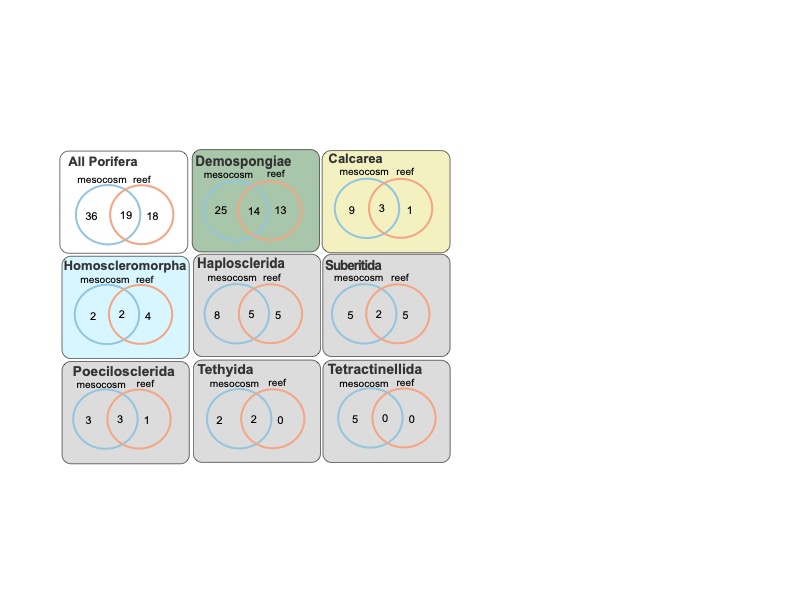


**Supplementary Figure S2.** Number of shared OTUs between modified mesocosm and reef ARMS for sponge classes Calcarea (yellow), Homoscleromorpha (blue) and Demospongiae (green). Orders within the class Demospongiae are listed in grey.


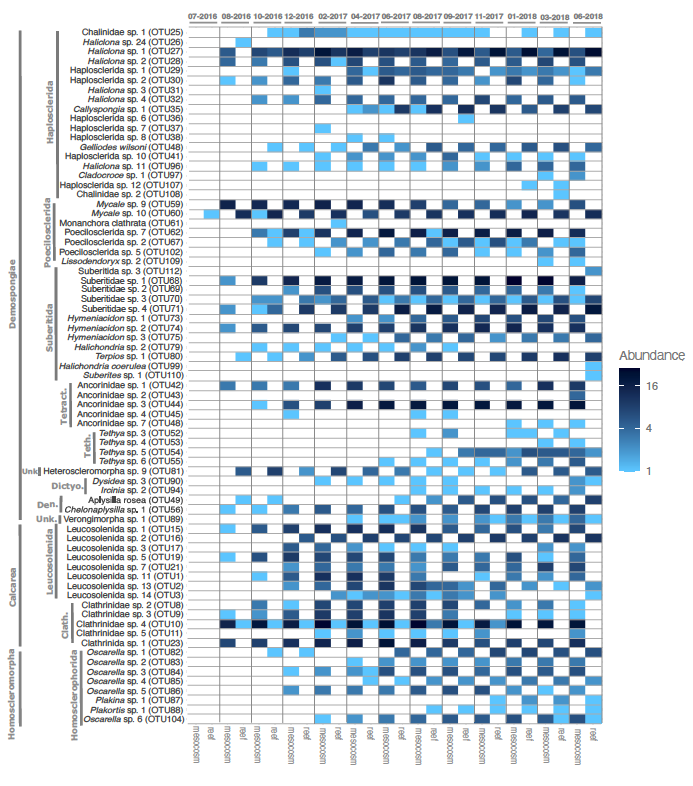


**Supplementary Figure S3** Temporal abundance of 73 cryptic sponge species on mesocosm and reef ARMS in Moku o Loʽe, Kāneʽohe Bay. Abundance is based on the sum of the presence/absence of each species per habitat. Each habitat has 6 ARMS with 3 plates each, including side (top/bottom of plate), for a total of 36 possibilities per species. ARMS were monitored every two months throughout a two-year period. Sponge classes and orders are indicated adjacent to each OTU name and number.

**Supplementary Figure S4** Proportion of colonial and solitary sponge growth morphologies of the 73 OTUs present on reef and mesocosm ARMS.

**Supplementary Figure S5**. Boxplot of cumulative (a) observed diversity and (b) abundance of sponges collected bimonthly during 2 years in mesocosm and reef ARMS. Significant t-test results are indicated by an * with respective p-values. Results from all t-test are presented in Table 1. Bars are median values for each sponge group; boxes show the upper and lower quartile; whiskers indicate the least and greatest value, excluding outliers. Mean values are shown as black circles and outliers as red circles.


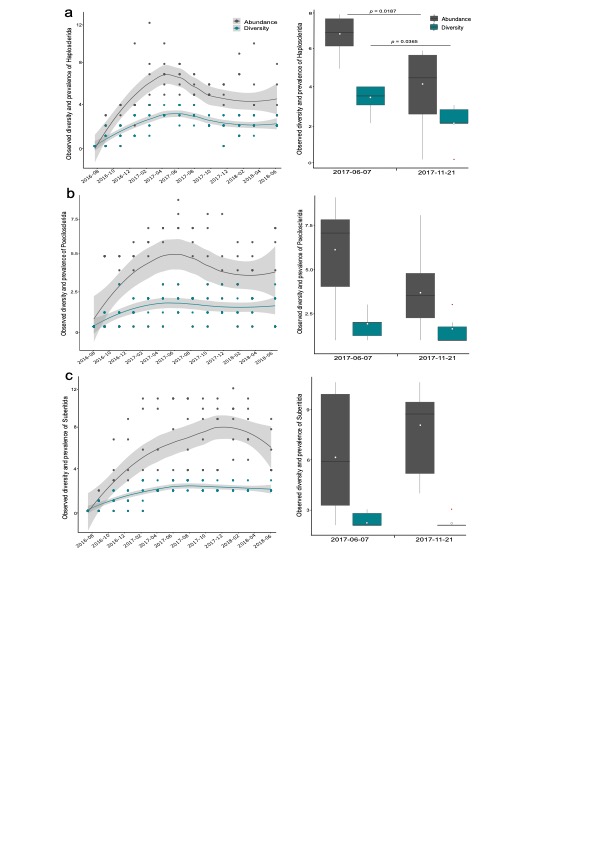


**Supplementary Figure S6** Temporal recruitment of sponge OTUs and abundance settling on mesocosm ARMS for the most abundant orders within class Demospongiae (a) Haplosclerida, (b) Poecilosclerida, and (c) Suberitida. The left panel shows temporal recruitment, monitored bimonthly for approximately two years (July 2016 through June 2018). Peak diversity and abundance were observed at 11 months (June 2016) and was lowest by 16 months (November 2016) thereafter. Significant Kruskal Wallis test results of declines in diversity and abundance are reported for class order (a) Haplosclerida. Line graphs are presented with lower and upper pointwise confidence intervals. Boxplots show median values as bars for each habitat; boxes show the upper and lower quartile; whiskers indicate the least and greatest value, excluding outliers. Mean values are shown as open circles and red circles are outliers.

**Supplementary Figure S7** Sequential images of the same mesocosm ARMS plate showing recruitment of *Haliclona* sp. 1 (OTU 27) indicated by white arrows on top right panel (at 10 months) disappearing gradually through a five-month period.


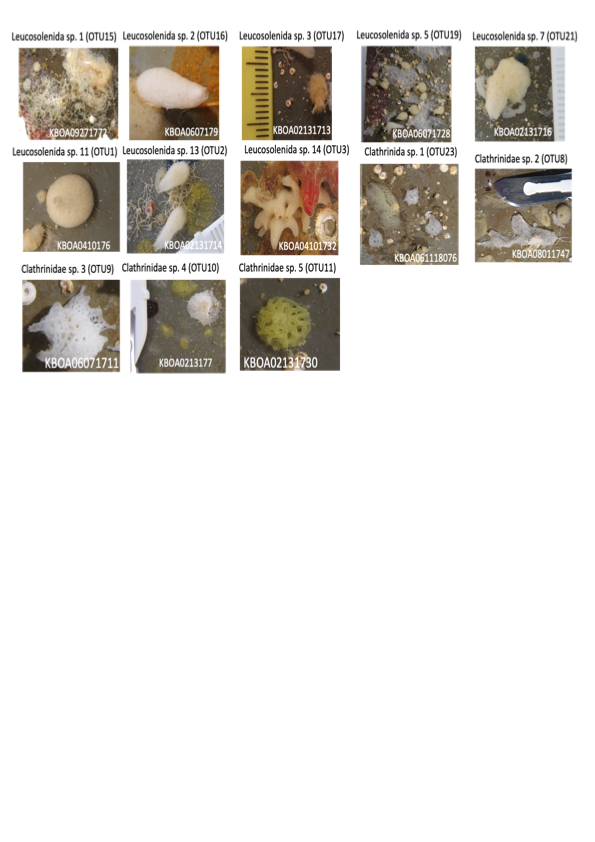


**Supplementary Figure S8** *In situ* images of the 13 calcareous OTUs growing on reef and mesocosm ARMS mostly showing solitary growth morphologies.
